# Spatial suitability evaluation and layout optimization of emergency shelter: A case study in Tianhe District of Guangzhou City

**DOI:** 10.1016/j.heliyon.2024.e41122

**Published:** 2024-12-12

**Authors:** Bo Tang, Zongyuan Li, Yinzhong Chen

**Affiliations:** Guangzhou Xinhua University, School of Resources and Planning, Guangzhou, 510520, China

**Keywords:** Emergency shelter, Suitability evaluation, Grey correlation method, Layout optimization, Tianhe district

## Abstract

Emergency shelters are multifunctional spaces that provide safe refuge, essential life protection, and rescue command for residents in case of urban disaster. These shelters constitute crucial components of urban public safety. This study, with Tianhe District in Guangzhou City as a case study, used data from emergency evacuation sites and other socio-economic sources to construct an evaluation system for spatial suitability evaluation and layout optimization of emergency shelters. This paper utilized from GIS-based methods including Grey Correlation Method (GCM) and Entropy Weight Method (EWM), the research process of “suitability evaluation —evacuation gap analysis—site selection—site optimization”is established, and the suitability evaluation results are calculated from the combination of effectiveness, accessibility and safety; based on the principles of fairness, accessibility, safety and feasibility, combined with the analysis of the current situation of land use and evacuation demand, the layout of urban emergency evacuation sites is adjusted. Finally, the spatial layout optimization plan for emergency shelters in Tianhe District was obtained. The results show that: (1) the overall suitability of the 50 emergency shelters in Tianhe District is favorable: 21 shelters are classified as “suitable,” 25 shelters as “slightly suitable,” and four as “unsuitable.” (2) Most significant evacuation gaps are located in the peripheral areas of Tianhe District. By adding five new evacuation sites to optimize the existing layout, the evacuation coverage increased from 68.4 % to 85.2 %. Consequently, the number of people in need of evacuation decreased from 387,900 to 80,100, so the reasonable allocation of evacuation space has been significantly improved. At the same time, the optimised scheme increases the density of emergency shelters and reduces the overlap of services to some extent. Evaluating the appropriateness and optimizing the spatial layout of urban emergency shelters can enhance disaster prevention and response capacity in megacities. This approach is of great practical value for the construction of resilient cities and the implementation of sustainable development strategies.

## 1 Introduction

Urban safety is the touchstone of urban construction. The capacity for comprehensive disaster prevention and control is becoming an extensive measure of the overall function of the city and its safety and defense capabilities (Wang, 2003) [1]. As a hub for population and socio-economic activities, the city represents a complex system of nature-economy-society. The ability to cope with disasters and recover from them is crucial for sustainable development. It is essential to establish a science-based and effective comprehensive urban disaster prevention and emergency response system and planning program in line with national development strategies and policies (Fang & Wang, 2015) [2]. With the ever-accelerating urbanization, potential risks such as natural disasters and public health events present dynamic, complex, and uncertain challenges that threaten sustainable development. Considerable attention is now being paid to the question of how to build a harmonious, healthy, green, safe, and livable city (Zhai & Xia, 2021) [3]. As urban disaster prevention planning evolves towards “evolutionary resilience,” there is an urgent need to apply theories and research methods from complex networks. This involves full consideration of the interrelationships and mechanisms between various disasters. It also entails in-depth research on the intrinsic correlation structures of complex urban systems and their developmental and evolutionary patterns (Ming et al., 2013) [4]. The aim is to actively build a safe and resilient comprehensive disaster prevention and control system.

As one of the megacities in South China, Guangzhou is densely populated with high mobility, economically developed, and with high building density, and faces greater risks of natural disasters, accidental catastrophes, and public health events in the context of global climate change and socio-economic development. In 2006, Guangzhou successively constructed a number of well-equipped and well-functioning earthquake emergency evacuation sites. In 2019, the “Guangzhou Territorial In 2019, the “Guangzhou Spatial Master Plan (2018–2035)" proposed the construction of a safe and resilient city, which put forward new requirements for Guangzhou's future spatial planning. In 2021, Guangzhou's “14th Five-Year Plan” proposed to firmly establish the concept of safety development, build a resilient city, and strengthen the intrinsic safety. The “safe development model city” and resilient city will also become new goals and policies for Guangzhou's future development.

Additionally, Guangzhou is in a critical seismic surveillance and defense zone in China. In areas such as Tianhe District, Yuexiu District, and Liwan District, the seismic intensity is classified as degree Ⅶ. There are 362 emergency shelters of various types in Guangzhou, with a total effective area of 3,473,500 square meters. Still, the service coverage rate of emergency shelters in Tianhe District is only 23.97 %, indicating a sizeable sheltering gap. In the event of a disaster, this gap will bring about incalculable loss of life and property. Therefore, urban planning and construction should be more proactive, with greater attention paid to the construction of refuge and disaster prevention facilities.

## 2 Review of previous studies

Urban emergency shelters are a concentrated expression and critical component of comprehensive urban disaster prevention planning intended primarily for residents to avoid disasters or for rescue departments to focus on the critical rescue place (Zhao et al., 2014) [5]. They can significantly enhance the efficiency of urban response to disasters and security emergency events and enable self-help and repair capabilities. This is crucial to the sustainable development of urban society and the economy (Marjolein et al., 2017) [6]. In recent years, emergency shelters have increased dramatically in number and scale, and scholars at home and abroad have accumulated a very large number of results based on the target population of emergency shelters, functional design and conversion of emergency shelters, and management of emergency shelters. Meanwhile, field research, quantitative measurements, empirical analyses and big data platforms are used to discuss the evacuation capacity, spatial accessibility, evacuation paths and planning of emergency shelters. Scholars at home and abroad emphasise the theoretical basis of multidisciplinary intersection, and try to conduct comprehensive research on emergency sheltering places in a multi-angle, multi-scale and multi-method research approach. It is mainly reflected in two major aspects.

### 2.1 Spatial layout and functions of emergency shelters

Since the 21st century, research on emergency shelters has started to evolve from single issues and events to integrated systems. The main focus is on the research process of emergency shelter location selection, spatial layout, functional design, and daily management focusing on the variety of field research types, data statistical rigour and information visualization. The research methodology has shifted from qualitative exploration of methodological models to the development of power mechanisms and information systems, and rich results have been achieved in the prediction of evacuation capacity, spatial accessibility, and evacuation routes of emergency shelters. Bandana et al. (2008) evaluated the suitability and service functions of emergency shelters in Florida, USA, using weighted linear combination and GIS [7]. Evacuation capacity prediction is essential for the spatial siting, construction, and management of emergency evacuation sites. Chou et al. (2013) established the Emergency Shelter Capacity Measurement System (ESCMS) was established in conjunction with the Earthquake Loss Estimation System (TELES) software, providing valuable references for relevant organizations to formulate earthquake prevention and disaster reduction policies [8]; Chen et al. (2016) developed a prediction model for the proportion of population evacuated from earthquake hazards based on the ratio of building seismic hazard evacuation rates to the categorized area ratio of building vulnerable structures [9]. Anhorn et al. (2015) focused on the accessibility analysis of emergency evacuation sites from the perspective of geographical distance, the Open Space Suitability Index (OSSI) for contingency planning and placement of shelter in the immediate aftermath of a disaster was introduced [10]. Tetsuya et al. (2019) conducted a quantitative suitability analysis of emergency shelter configuration after an earthquake in Japan based on GIS, statistical methods, and publicly available data related to population and emergency shelters [11]. Yao et al. (2021) selected public open spaces in Greater Victoria as the study area and used the multi-criteria TOPSIS evaluation model to comprehensively and quantitatively evaluate the safety, accessibility, and usability of these spaces, finding that the distribution of open spaces failed to match the dynamics of the population distribution [12]. Liu et al. (2017). used five indicators of effectiveness and accessibility, such as the number of people that can be accommodated in the emergency shelter, the closest distance to medical stations, and the closest distance to significant sources of danger. They determined the weight of the indicators based on the entropy method and integrated it with the grey correlation analysis method to evaluate the suitability of 79 emergency shelters in the Longgang District of Shenzhen [13]. Wang et al. (2022) constructed an evaluation index system for the spatial distribution of urban emergency shelters based on the five criteria of effectiveness, accessibility, safety, appropriateness, and fairness. It was combined with hierarchical analysis method (AHP), Criteria Importance Though Intercrieria Correlation (CRITIC), the optimal weight coefficient solution method based on the maximum deviation sum of squares theory, and fuzzy optimization theory. They carried out spatial optimization of emergency shelters in Shanghai from both individual and regional scales [14].

### 2.2 Spatial selection and optimization of emergency shelters

Emergency shelters are often affected by multiple factors such as socio-economics, land planning and government management, resulting in problems such as inappropriate siting, unbalanced spatial distribution and limited-service functions. To optimize the layout of the emergency shelters, it is important to combine them with the level of disaster risk, with a focus on scientific planning layout, and siting, which have become central to academic research (Tang et al., 2017) [15]. Most scholars combine the use of geographic information systems to construct spatial siting models for emergency shelters, such as multi-criteria mathematical models, decision support systems, and comprehensive indicator systems, and optimize the functions in terms of regional layout, capacity measurement, evacuation paths, service scope, effectiveness, and responsibility zoning. For example, Toregas et al. (1971) proposed the siting problem for emergency service facilities to determine the minimum number of facilities required to cover the maximum range of emergency service demand points [16]. Wu et al. (2010) used the Voronoi polygon algorithm to study the spatial optimization and configuration status of emergency shelters in Beijing. Their results show a gap in the construction of emergency shelters in Beijing, along with an unbalanced spatial distribution [17]; Zhou et al. (2014) conducted a service radius buffer analysis, spatial superposition analysis, and accessibility analysis of emergency refuge places in Beijing. Using the spatial analysis module of GIS, they assessed the effectiveness of these shelters, concluding that the urban area of Beijing generally cannot meet the residents' demand for refuge [18]; Trivedi et al. (2017) proposed a hybrid algorithm based on the AHP for efficient management of site selection and relocation projects. The aim was to build an automatic emergency shelter layout model. The proposed minimizes distance, risk, the number of sites and uncovered demand [19]; Li et al. (2018) selected factors such as urban population density and the range of fixed emergency shelter service areas, with GIS and AHP used to study the emergency response capacity of the city after an earthquake [20].

### 2.3 Literature summary

In summary, research by Chinese and foreign experts on the evaluation of the suitability and layout optimization of emergency shelters has reached a mature stage. The selection of evaluation indexes has become increasingly rigorous and systematic, and the evaluation methodology has experienced a shift from qualitative to quantitative, and from a single perspective to a comprehensive paradigm (Tang & Qiu, 2019) [21]. Although different scholars have applied many methods for the evaluation of emergency shelters, there is still room for deeper exploration in the he data integration、selection of evaluation indexes and quantitative processing of indexes. Furthermore, community as the smallest administrative unit of Chinese urban management, but bear the largest unit of the whole social body, encompassing geographic location, demographic characteristics, traffic guidelines, ecological environment and many other aspects. Meanwhile, as the basic unit of urban space and social organisation, community disaster prevention is a fundamental part of urban disaster prevention and control. Micro-scale research on the spatial pattern and optimization of emergency shelters is becoming more and more important, which is also conducive to the improvement of provincial and municipal emergency shelter planning. Therefore, how to design the spatial layout and optimization of emergency shelter sites with multi-source data, refinement and high efficiency is the focus of future research, which is of great significance to the urban public safety construction system.

Based on the use of multi-source geospatial and socio-economic data, this study attempts to conduct a comprehensive analysis of urban emergency shelters from a neighbourhood perspective (microscopic scale) by using quantitative models and spatial analyses in multiple dimensions such as such as “suitability evaluation—evacuation gap analysis—site selection—spatial optimization”, so as to provide necessary theoretical support for the planning and layout of emergency shelters in the Tianhe District of Guangzhou and related cities across the country. This study selects nine indicators, such as shelter capacity, open space ratio, road density, etc., from the three major guidelines of effectiveness, accessibility, and safety. GIS technology is used to evaluate the appropriateness of built parks, plazas, stadiums, and other emergency evacuation locations in Tianhe District. Based on the four major principles, this study selects seven indicators, such as the demand for evacuation and the current land use. It conducts a spatial layout optimization to address the evacuation gap in Tianhe District. Besides, this study provides relevant suggestions for spatial layout optimization of emergency shelters and the construction. More importantly, it also provides new ideas for Guangzhou to adapt to the new development pattern, cope with public security, and optimize the land space, and provides policy reference for the construction of “resilient city".

## 3 Study area and data sources

### 3.1 Study area

**Fig. 1:**
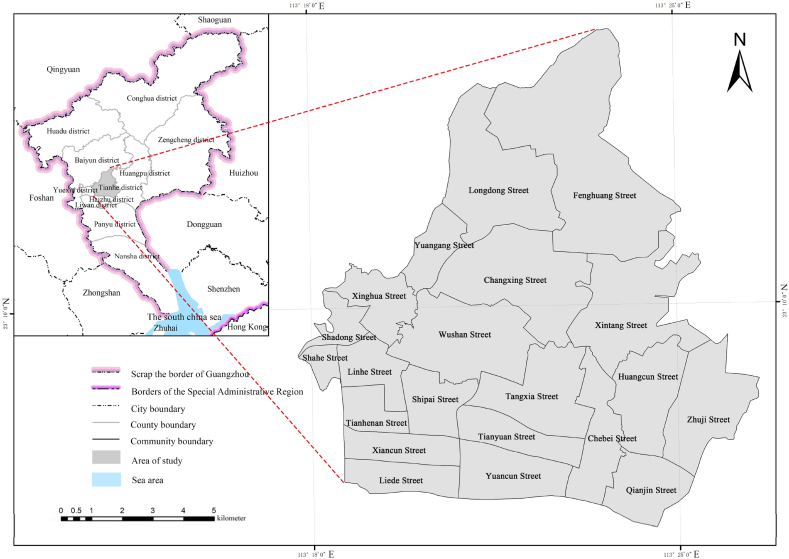
Study area.

Tianhe District has a total administrative area of 137.38 square kilometers. It is located in the eastern part of the old urban area of Guangzhou, the city's central business district (Fig. 1). According to the data released by the Seventh Population Census in 2020, the resident population of Tianhe District is 224,182,626, and the density of the resident population is 23,273/km^2^, which is the second largest in terms of the density of resident population in Guangzhou. Tianhe District is the city center of Guangzhou and is the central hub for transport in the city, the district has a concentration of transport resources and a multi-level urban transport system, including a metro, bus rapid transit (BRT) system, and railway station. Tianhe District is located in the south-central Guangdong region of the low hills and the Pearl River Delta in the transition zone; geomorphological types, geological and environmental conditions are complex and is located in the southern subtropical monsoon climate zone, the concentration of rainfall in the summer, prone to convective solid weather caused by local rainstorms, thunderstorms, coupled with human engineering activities in recent years the formation of hardening of the ground and other impacts, resulting in disasters, prone to, and frequently occurring. Although there have been no significant earthquake disasters in recent years, there are active rupture zones and seismic zones in and around the area within 200 km, which, coupled with the concentration of commercial and residential areas in Tianhe, makes the area prone to secondary disasters.

### 3.2 Data source

**Fig. 2:**
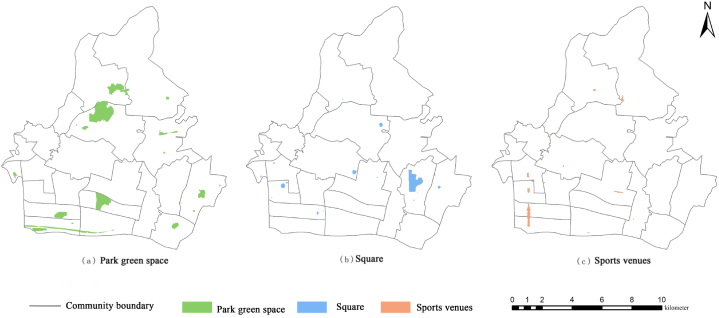
Distribution of different types of emergency shelters in Tianhe District.

The data on emergency shelters are obtained by searching parks, squares, and sports venues in the Tianhe District of Guangzhou City by Gaode Area of Interest (AOI). According to the Guangzhou local standard “Design Code for Emergency Sheltering Places,” the effective area of the minor emergency shelter site should be more than 2000 m^2^ (0.2 hm^2^). The existing sites are screened according to this scale, removing those that do not comply with the code and then determining a total of 50 emergency sheltering places that satisfy the conditions, among which there are 20 parks, 12 plazas, 18 stadiums, as shown in Fig. 2 and Table 1. The total area of emergency shelters in Tianhe District is 599.04 hm^2^, and the total effective shelter area is 363.12 hm^2^. The total effective evacuation area of parks is 264.76 hm^2^, accounting for 73 % of the total effective evacuation area of Tianhe District; the total effective evacuation area of squares is 39.50 hm^2^; and the total effective evacuation area of stadiums is 60.86 hm^2^. Other relevant data are shown Table 2.

**Table 1:** Information on emergency shelters in Tianhe District.

Number	Name	Total area/hm^2^	Effective sheltered area/hm^2^	population capacity/million people	Number	Name	Total area/hm^2^	Effective sheltered area/hm^2^	population capacity million/people
1	South China Botanical Garden	158	94.8	63.20	26	Longdong Culture Square	2	1.6	1.07
2	Tianhe Park	70	42	28.00	27	Sports East Leisure Belt Plaza	1.08	0.86	0.57
3	Guangdong Tree Park	46.62	27.97	18.65	28	South China University of Technology South Gate Square	0.88	0.7	0.47
4	Linjiang Ribbon Park	29.48	17.69	11.79	29	Linhe Street Derong Community Culture and Sports Square	0.65	0.52	0.35
5	Zhujiang Park	28	16.8	11.20	30	Pearl River Main Stream Plaza	0.64	0.51	0.34
6	Tianhe Children's Park	20.6	12.36	8.24	31	Yuan Gang Cultural Square	0.4	0.32	0.21
7	Haixinsha Asian Games Park	16.59	9.95	6.63	32	Chebei Square	0.31	0.24	0.16
8	North Coast Cultural Park	13.8	8.28	5.52	33	Guangdong Olympic Sports Centre	82.09	41.04	27.36
9	Yangtao Park	13.41	8.05	5.37	34	Guangzhou Tianhe Sports Centre Stadium	7.02	3.51	2.34
10	Tianhe Wetland Park	11.66	7	4.67	35	Hui Jing Golf Club	6.02	3.01	2.01
11	Yinpai Ling Park	11.16	6.7	4.47	36	Lanpeng Golf Training Centre	4.95	2.48	1.65
12	Changjian Park	7.4	4.44	2.96	37	Guangzhou Players Karting Club	2.58	2.58	1.72
13	Nineteenth Route Army Songhu Anti-Japanese War Memorial Park	5.84	3.5	2.33	38	Seventy-two Par Golf Club	2.32	2.32	1.55
14	Gaotangshi Park	3.16	1.9	1.27	39	Sports East Football Stadium	1.07	1.07	0.71
15	Zhucun Beggar's Nest	1.54	0.92	0.61	40	Bayi Football Team	1.02	1.02	0.68
16	Xintang Sai Wai Park	1.17	0.7	0.47	41	Lifang Sports Wisdom Football Park	0.69	0.69	0.46
17	Jiangyueyuan Small Playground	1	0.6	0.40	42	Nanqing Football Club	0.31	0.31	0.21
18	Qishan Park	0.84	0.5	0.33	43	MVP Football Pitch (Zhujiang New Town Shop)	0.28	0.28	0.19
19	Chang Janet New Village Garden	0.53	0.32	0.21	44	Tongdong Football Ground	0.24	0.24	0.16
20	Huangcun Park	0.47	0.28	0.19	45	Tianhe Village Football Pitch	0.22	0.22	0.15
21	Huacheng Plaza	24.81	19.84	13.23	46	Utopia Vitality Base Vitality Football Pitch	0.21	0.21	0.14
22	Tianhe Sports Centre South Square	4.18	3.34	2.23	47	Back Garden Tennis Club	0.93	0.93	0.62
23	Tongde Flower Garden - Culture Square	4.15	3.32	2.21	48	Juntai Tennis Centre	0.21	0.21	0.14
24	Furnace Mountain Forest Park North Square	4	3.2	2.13	49	Guangzhi Sports Basketball Centre	0.44	0.44	0.29
25	Guangzhou East Railway Station Square	3.77	3.01	2.01	50	Fei Meng Basketball Park (Botanical Garden)	0.31	0.31	0.21

**Table 2:** Other relevant data.

Datatypes	Time	Source
Road network	2021	https://www.openstreetmap.org/
Land Use Type	2021	Environmental Systems Research Institute (ESRI)
Population	2020	The Seventh China Population Census Bulletin
DEM	2021	https://data.bris.ac.uk/data/dataset/25wfy0f9ukoge2gs7a5mqpq2j7
Building	2021	Building Height Estimation Model for China at 10m Resolution (CNBH-10m) (Wu et al., 2023) [22]、Microsoft Building Profile Dataset
Facility Sites	2021	https://lbs.amap.com/

## 4 Methods

**Fig. 3:**
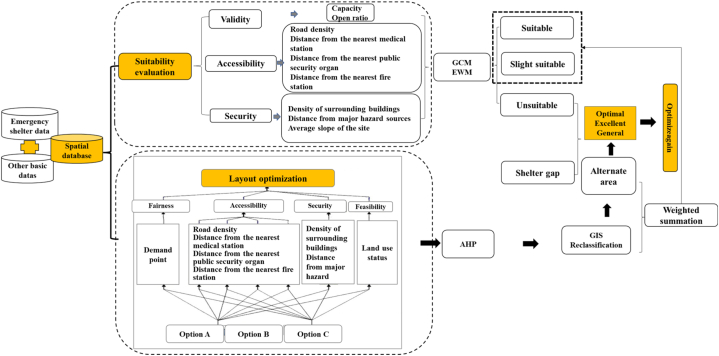
The flowchart of the methodology.

Based on the design specifications for emergency shelters, and with the aid of 3S technology and mathematical modeling method, the research focuses on two main aspects: evaluation of the suitability of emergency shelters and optimization of spatial layout. The suitability evaluation is based on three criteria: effectiveness, accessibility, and safety. Nine index factors are selected to establish the evaluation index system for the suitability of urban emergency shelters. The suitability evaluation results are calculated using a combination of GCM and EWM; based on the principles of fairness, accessibility, safety, and feasibility, along with an analysis of the current land use and evacuation demand, AHP and GIS weighting methods are used to evaluate the suitability of emergency shelters. The emergency evacuation sites are appropriately increased or expanded to obtain a fair and reasonable optimization plan for the spatial layout of the emergency evacuation sites in Tianhe District. Fig. 3 shows the flowchart of the methodology.

### 4.1 Suitability evaluation model

#### 4.1.1 Construction of evaluation system

The index system method is one of the more scientific methods for suitability evaluation. It has been widely used in land use and other fields and has gradually extended to disaster prevention and mitigation planning (Shi et al., 2007; Huang et al., 2008) [23,24]. The influencing factors of the suitability of emergency shelters are complex, and how to scientifically construct the evaluation index system has become the key to the suitability evaluation of emergency shelters (Tie & Tang, 2005) [25]. From the three aspects of validity, accessibility, and safety, this paper selects nine indicators, such as population, road density, and slope, to construct the suitability evaluation index system for urban emergency shelters (Table 3). Positive indicators positively affect the suitability of emergency shelters; the higher the evaluation value, the higher the suitability; negative indicators hurt the suitability evaluation of emergency shelters; the higher the evaluation value, the lower the suitability (Lv et al., 2023) [26].

**Table 3:** Suitability evaluation index system.

Indicator criteria layer	Evaluation index	Target Vector
Validity	Open space ratio	Positive
	The number of people it can accommodate	Positive
Accessibility	Road density	Positive
	Distance from the nearest medical station	Negative
	Distance from the nearest fire station	Negative
	Distance from the nearest public security organ	Negative
Security	Slope	Negative
	Height of surrounding buildings	Negative
	Distance from significant hazard sources	Positive

#### 4.1.2 GCM

The grey correlation system theory can comprehensively analyze the system's multi-factor interaction (Liu et al., 2005) [27]. And GCM does not need many samples and distribution laws (Yu & Fu, 2004) [28]. The specific steps are as follows.

1 Equation (1) represents the optimal sequence for determining the suitability evaluation of emergency shelter locations:

$$X_{0}=\left\{X_{0}\left(1\right),X_{0}\left(2\right),\ldots ,X_{0}\left(n\right)\right\}$$

$$X_{1}=\left\{X_{1}\left(1\right),X_{1}\left(2\right),\ldots ,X_{1}\left(n\right)\right\}$$

$$X_{2}=\left\{X_{2}\left(1\right),X_{2}\left(2\right),\ldots ,X_{2}\left(n\right)\right\}$$

(1) $$X_{i}=\left\{X_{i}\left(1\right),X_{i}\left(2\right),\ldots ,X_{i}\left(n\right)\right\}$$

$$X_{m}=\left\{X_{m}\left(1\right),X_{m}\left(2\right),\ldots ,X_{m}\left(n\right)\right\}$$

in the formula,i = 1,2,3, …,m.

2 Indicator dimensionless: As shown in equation (2) 、(3) and (4), according to the nature of the evaluation indicators, the upper limit effect measure and the lower limit effect measure are applied to the positive and negative indicators, respectively.

The upper limit effect measure:

(2) $$\begin{array}{c} r_{ij}=\frac{X_{ij}}{\max\left(X_{ij}\right)} \end{array}$$

The lower limit effect measure:

(3) $$\begin{array}{c} r_{ij}=\frac{\min\left(X_{ij}\right)}{X_{ij}} \end{array}$$

The dimensionless treatment based on the different properties of each indicator yields a homogenized series, i.e.

$$X_{0}^{\prime }=\left\{X_{0}^{\prime }\left(1\right),X_{0}^{\prime }\left(2\right),\ldots ,X_{0}^{\prime }\left(n\right)\right\}$$

$$X_{1}^{\prime }=\left\{X_{1}^{\prime }\left(1\right),X_{1}^{\prime }\left(2\right),\ldots ,X_{1}^{\prime }\left(n\right)\right\}$$

$$X_{2}^{\prime }=\left\{X_{2}^{\prime }\left(1\right),X_{2}^{\prime }\left(2\right),\ldots ,X_{2}^{\prime }\left(n\right)\right\}$$

(4) $$X_{i}^{\prime }=\left\{X_{i}^{\prime }\left(1\right),X_{i}^{\prime }\left(2\right),\ldots ,X_{i}^{\prime }\left(n\right)\right\}$$

$$X_{m}^{\prime }=\left\{X_{m}^{\prime }\left(1\right),X_{m}^{\prime }\left(2\right),\ldots ,X_{m}^{\prime }\left(n\right)\right\}$$

in the formula,i = 1,2,3, …,m.

3 Calculate the grey correlation coefficient (Equation (5)and (6)):

Firstly, the absolute difference Δ (k) is calculated as follows:

(5) $$\begin{array}{c} \Delta_{0i}\left(k\right)=\left|X_{0}^{\prime }\left(k\right)‐X_{i}^{\prime }\left(k\right)\right|\; \end{array}$$

In the formula, $\left\{X_{0}^{\prime }\left(k\right)\right\}$ and $\left\{X_{i}^{\prime }\left(k\right)\right\}$ is the absolute value of the k_th_ term at point i,k = 1,2,3, …,n。

Calculate the maximum difference between the two levels, $\max_{i}\max_{k}\Delta_{i}\left(k\right)$ and the minimum difference between the two levels, $\min_{i}\min_{k}\Delta_{i}\left(k\right)$;

Secondly, the grey correlation coefficient was calculated as follows:

(6) $$\begin{array}{c} r\left(X_{0}\left(k\right),X_{0}\left(k\right)\right)=\frac{\min_{i}\min_{k}\Delta_{i}\left(k\right)+\xi \max_{i}\max_{k}\Delta_{i}\left(k\right)}{\Delta_{i}\left(k\right)+\xi \max_{i}\max_{k}\Delta_{i}\left(k\right)} \end{array}$$

i = 1,2,3, …,m; k = 1,2,3, …,n; ξ is the resolution factor, ξϵ(01] The empirical value of ξ equal to 0.5 is mostly used in related studies [24,25],Therefore, in this paper ξ takes the value 0.5。

4 Calculation of weighted correlation: As shown in Equation (7), in calculating the correlation, a weighted approach is used, and the sum of the product of the grey correlation coefficient $r_{ij}$ of each indicator and the weight $W_{ij}$ of each indicator is used as the final evaluation result. The following formula can be used for calculation:

(7) $$\begin{array}{c} R_{i}=\sum_{j=1}^{n}r\left(X_{0}\left(k\right),X_{i}\left(k\right)\right)W_{j} \end{array}$$

In the formula, $W_{j}$ is the weight value; the larger $R_{i}$, the closer the program is to the best program,i.e., the better the suitability; on the contrary, the worse it is. Sorting $R_{i}$ in a particular order, we can get the evaluation result of the appropriateness of the emergency shelter.

#### 4.1.3 EWM

In this paper, the weights of indicators are determined by using EWM, EWM is a kind of information quantity weight method (Zhang et al., 2010) [29]. As shown in equations (10), (11), (12), (13), (8), (9), the specific calculation steps are as follows:

1 Data standardisation processing:

Positive indicators:

(8) $$\begin{array}{c} S_{ij}^{\prime }=\frac{S_{ij}-\min\left\{S_{j}\right\}}{\max\left\{S_{j}\right\}-\min\left\{S_{j}\right\}} \end{array}$$

Negative indicators:

(9) $$\begin{array}{c} S_{ij}^{\prime }=\frac{\max\left\{S_{j}\right\}-S_{ij}}{\max\left\{S_{j}\right\}-\min\left\{S_{j}\right\}} \end{array}$$

2 Calculate the contribution of the i th programme for the j th indicator, $P_{ij}$:

(10) $$\begin{array}{c} \;P_{\text{ij}}=\frac{S_{\text{ij}}}{\sum_{i=1}^{m}S_{\text{ij}}}\; \end{array}$$

3 Calculate the information entropy of the indicator $E_{j}$ :

(11) $$\begin{array}{c} E_{j}=-\ln\;{\left(n\right)}^{-1}\sum_{i=1}^{m}P_{ij}\;\ln\left(P_{ij}\right) \end{array}$$

④Calculate the information entropy redundancy $D_{j}$:

(12) $$\begin{array}{c} D_{j}=1-E_{j} \end{array}$$

⑤Calculate the indicator weights $W_{j}$:

(13) $$\begin{array}{c} W_{j}=\frac{D_{j}}{\sum_{j=1}^{m}D_{j}} \end{array}$$

### 4.2 Spatial layout optimization model

#### 4.2.1 Layout optimization principle of shelter

##### 4.2.1.1 Accessibility

The establishment of emergency shelters also needs more service functions, and the high road density and the shelter gap close to the service facilities are more conducive to site selection.

##### 4.2.1.2 Safety

New shelters should avoid secondary disasters from the source and be built in safer places. According to the local standard Code for Design of Emergency Shelter in Guangzhou, the slope of the emergency functional area shall be less than 8 %, and the distance from the significant secondary fire or explosion hazard sources such as flammable and explosive factory warehouses, gas supply plants and gas storage stations shall not be less than 1000 m. Therefore, the location of new shelters should remove these areas that do not meet the site's requirements. Combined with the height data of the surrounding buildings, the lower the location, the better.

##### 4.2.1.3 Fairness

After a disaster, the fastest way to reach the shelter is to ensure the safety of residents, so the layout of emergency shelters should be evenly distributed in the city, and its construction should adhere to the principle of balanced layout so that each resident can choose the nearest shelter fairly and to the greatest extent. Secondly, but also a reasonable layout, the demand for refuge in places with a large population and shelters should be set up in large numbers, on a large scale. For sparsely populated or unpopulated areas, the layout should be reduced. The fairness of the layout optimization in this study is reflected in the following aspects: the relocation of unsuitable shelters and shelter gaps, the addition of new shelters, and the addition of new shelters for residents outside the service area as far as possible. Among them, the center of the building is taken as each residential unit, and the population of each district is averaged down to each residential unit. The more concentrated the residential units in the shelter gap are, the more residents are, and the more significant the impact on the location of new shelters.

##### 4.2.1.4 Feasibility

Water, farmland, and other land types are not suitable for constructing new shelters, so we should consider the current land use situation to analyze the feasibility of site selection.

#### 4.2.2 Layout optimization method of emergency shelter

In this paper, the spatial layout optimization scheme of emergency shelters is mainly transported to the tool module of GIS, and the above optimization principles are assigned to the indicators through AHP. The Euclidean distance of each indicator is calculated, and finally, the re-classified indicators are computed using a grid calculator to find the optimal location of the shelter gap.

##### 4.2.2.1 AHP

AHP is a hierarchical and structural decision-making method for analyzing multiple schemes and index systems, which has the advantages of applicability, conciseness, effectiveness, and systematicness (Ji et al., 2021) [30]. Many scholars at home and abroad have applied the analytic hierarchy process to evaluate emergency shelters (James, 2008) [31]. According to the principle and calculation process of the analytic hierarchy process, equation (14) is used to calculate the comprehensive disaster reduction capacity of the emergency shelter:

(14) $$\begin{array}{c} P_{i}=C_{i}W_{i} \end{array}$$

Among $P_{i}$ is a comprehensive index of the site suitability of a particular refuge-gap $C_{i}$ It is the comprehensive score of the fairness, accessibility, security and feasibility indicators of the refuge gap. $W_{i}$ is the weight value of the refuge gap analytic hierarchy process, and *i* is the evaluation element. Among $P_{i}$ The higher the value is, the better the disaster reduction ability of the plot is.

##### 4.2.2.2 GIS analysis method

① Network analysis: Network analysis generally includes finding the best route, determining the nearest public facilities, and creating service areas. Often, when people flee, they do not flee at two points and one line, but according to the road network as an evacuation route, the shorter distance on the plane may not be the “nearest distance” in reality (Li et al., 2015) [32]. Therefore, when a disaster occurs, the evacuation routes of citizens and the rescue routes of rescue forces should be based on the road network, which is better than the traditional buffer analysis method.
② Overlay analysis: The overlay analysis function of GIS can aggregate two or more data to obtain new data. In this layout optimization study, the weighted sum method in the overlay analysis tool will be used to sum the raster data of the shelter gap to obtain the comprehensive index of location selection. $P_{i}$。

## 5 Results

### 5.1 Suitability evaluation of emergency shelter in Tianhe District

The network analysis and GIS spatial analysis module were used to obtain and process the data, constructed the data sequence of each evaluation index. According to the properties of the indicators, the indicator data are dimensionless and grey series are generated by equation (2)、(3). And the grey correlation coefficient of each evaluation index of the evaluation system was obtained (Table 4) according to equation (5) 、(6). Then, the entropy weight is calculated. It can be seen from Table 5 that the index of the number of populations that can be accommodated has the most significant weight among all the indexes, the index of the road density in the accessibility criterion has a more substantial weight, and the index of the distance from the significant hazard source in the safety criterion has a more considerable weight. Therefore, the adequate accommodation number of shelters is the most critical factor affecting the suitability evaluation results of emergency shelters in Tianhe District, and the accessibility index also has an essential contribution to the suitability evaluation results. Because the number of service facilities in Tianhe District is large, the coverage is good, and the distance from service facilities to emergency shelters is insignificant. Therefore, the weight of the distance between the accessibility index and the medical station, the fire station, and the public security organ is small, and the contribution is negligible. Finally, the comprehensive evaluation results of the suitability of 50 emergency shelters are obtained by combining the grey correlation coefficient and entropy weight (Table 6). According to the suitability evaluation results of shelters in Table 4, they are divided into the following three grades: suitable (R ≥ 0.45), relatively suitable (0.40 ≤ R < 0.45), and unsuitable (R < 0.40).

**Table 4:** Grey correlation coefficient.

Number	Open Space ratio	The number of people it can accommodate (People)	RoadDensity	Distance from the nearest medical station (m)	Recently Fire station distance	Distance from the nearest public security organ	Slope (°)	Height of surrounding buildings (m)	Distance from significant hazard sources (m)
1	0.555	1.000	0.455	0.338	0.349	0.361	0.337	0.563	0.341
2	0.555	0.472	0.586	0.353	0.382	0.371	0.341	0.478	0.337
3	0.555	0.414	0.465	0.342	0.357	0.360	0.337	0.561	0.344
4	0.555	0.380	0.751	0.340	0.369	0.406	0.343	0.457	0.369
5	0.555	0.377	0.437	0.340	0.360	0.379	0.351	0.445	0.530
6	0.555	0.364	0.454	0.342	0.349	0.384	0.338	0.634	0.368
7	0.555	0.358	0.442	0.341	0.373	0.460	0.342	0.501	0.439
8	0.555	0.353	0.418	0.341	0.374	0.380	0.341	0.585	0.565
9	0.555	0.353	0.376	0.339	0.372	0.390	0.371	0.548	0.424
10	0.555	0.350	0.416	0.350	1.000	0.373	0.348	0.543	0.396
11	0.555	0.349	0.370	0.339	0.367	0.373	0.334	0.619	0.338
12	0.555	0.343	0.346	0.352	0.356	0.417	0.345	0.599	0.461
13	0.555	0.341	0.394	0.352	0.379	1.000	0.344	0.508	0.489
14	0.555	0.337	0.492	0.335	0.402	0.349	0.340	0.709	0.585
15	0.555	0.335	0.416	0.339	0.352	0.479	0.402	0.659	0.420
16	0.555	0.334	0.372	0.337	0.361	0.387	0.373	0.775	0.533
17	0.555	0.334	0.495	0.338	0.360	0.398	0.417	0.444	0.563
18	0.555	0.334	0.366	0.339	0.345	0.353	0.342	0.681	0.938
19	0.555	0.333	0.373	0.342	0.358	0.392	0.350	0.715	0.423
20	0.555	0.333	0.613	0.338	0.361	0.469	0.395	0.680	0.472
21	0.714	0.387	0.668	0.346	0.391	0.470	0.395	0.434	0.466
22	0.714	0.341	0.816	0.353	0.381	0.435	0.362	0.587	0.526
23	0.714	0.341	0.458	0.354	0.566	0.524	0.345	0.480	0.380
24	0.714	0.340	0.863	0.339	0.351	0.391	0.336	0.553	0.357
25	0.714	0.340	0.370	0.342	0.582	0.450	0.346	0.443	0.384
26	0.714	0.336	0.684	1.000	0.352	0.399	0.348	0.699	0.388
27	0.714	0.337	1.000	0.343	0.434	0.407	0.419	0.450	0.469
28	0.714	0.336	0.543	0.348	0.404	0.404	0.347	0.566	0.442
29	0.714	0.335	0.461	0.345	0.389	0.612	0.387	0.443	0.489
30	0.714	0.335	0.778	0.347	0.391	0.421	0.342	0.650	0.689
31	0.714	0.334	0.756	0.341	0.363	0.457	0.343	0.632	0.372
32	0.714	0.334	0.370	0.340	0.410	0.372	0.340	0.753	0.374
33	0.499	0.468	0.484	0.337	0.358	0.370	0.348	0.627	0.338
34	0.499	0.341	0.447	0.344	0.402	0.386	0.391	0.637	0.461
35	0.499	0.340	0.420	0.338	0.364	0.361	0.338	0.471	0.598
36	0.499	0.339	0.371	0.336	0.357	0.350	0.335	0.560	1.000
37	1.000	0.339	0.373	0.337	0.350	0.355	0.343	0.735	0.441
38	1.000	0.338	0.368	0.339	0.353	0.359	0.780	0.797	0.426
39	1.000	0.338	0.565	0.347	0.427	0.402	0.364	0.454	0.481
40	1.000	0.338	0.370	0.342	0.406	0.428	0.657	0.469	0.433
41	1.000	0.336	0.340	0.335	0.347	0.349	0.337	0.633	0.476
42	1.000	0.334	0.360	0.340	0.366	0.393	0.400	0.693	0.465
43	1.000	0.334	0.370	0.340	0.361	0.367	0.378	0.645	0.372
44	1.000	0.334	0.371	0.340	0.397	0.387	0.367	0.871	0.428
45	1.000	0.334	0.366	0.359	0.420	0.381	0.344	0.537	0.708
46	1.000	0.334	0.604	0.338	0.362	0.395	0.446	0.638	0.452
47	1.000	0.337	0.410	0.349	0.375	0.594	0.344	0.428	0.497
48	1.000	0.334	0.371	0.340	0.353	0.360	1.000	1.000	0.409
49	1.000	0.335	0.516	0.342	0.361	0.420	0.409	0.751	0.339
50	1.000	0.334	0.505	0.347	0.351	0.373	0.403	0.614	0.382

**Table 5:** Weights of suitability evaluation index system.

Indicator criteria layer	Evaluation index	$W_{j}$
Validity	Open space ratio	0.121
	The number of people it can accommodate	0.501
Accessibility	Road density	0.110
	Distance from the nearest medical station	0.024
	Distance from the nearest fire station	0.033
	Distance from the nearest public security organ	0.040
Security	Slope	0.017
	Height of surrounding buildings	0.049
	Distance from significant hazard sources	0.105

**Table 6:** Evaluation results of the suitability of emergency shelters in Tianhe District.

Number	Place Name	R	Degree of suitability
1	South China Botanical Garden	0.721	Suitable
2	Tianhe Park	0.469	Suitable
3	Guangdong Tree Park	0.430	Slightly suitable
4	Linjiang Ribbon Park	0.444	Slightly suitable
5	Zhujiang Park	0.423	Slightly suitable
6	Tianhe Children's Park	0.410	Slightly suitable
7	Haixinsha Asian Games Park	0.410	Slightly suitable
8	North Coast Cultural Park	0.420	Slightly suitable
9	Yangtao Park	0.399	Unsuitable
10	Tianhe Wetland Park	0.419	Slightly suitable
11	Yinpai Ling Park	0.389	Unsuitable
12	Changjian Park	0.398	Unsuitable
13	Nineteenth Route Army Songhu Anti-Japanese War Memorial Park	0.424	Slightly suitable
14	Gaotangshi Park	0.427	Slightly suitable
15	Zhucun Beggar's Nest	0.403	Slightly suitable
16	Xintang Sai Wai Park	0.411	Slightly suitable
17	Jiangyueyuan Small Playground	0.413	Slightly suitable
18	Qishan Park	0.446	Slightly suitable
19	Chang Janet New Village Garden	0.396	Unsuitable
20	Huangcun Park	0.430	Slightly suitable
21	Huacheng Plaza	0.471	Appropriate
22	Tianhe Sports Centre South Square	0.475	Appropriate
23	Tongde Flower Garden - Culture Square	0.425	Slightly suitable
24	Furnace Mountain Forest Park North Square	0.458	Suitable
25	Guangzhou East Railway Station Square	0.411	Slightly suitable
26	Longdong Culture Square	0.463	Suitable
27	Sports East Leisure Belt Plaza	0.482	Suitable
28	South China University of Technology South Gate Square	0.432	Slightly suitable
29	Linhe Street Derong Community Culture and Sports Square	0.430	Slightly suitable
30	Pearl River Main Stream Plaza	0.488	Suitable
31	Yuan Gang Cultural Square	0.451	Suitable
32	Chebei Square	0.413	Slightly suitable
33	Guangdong Olympic Sports Centre	0.455	Slightly suitable
34	Guangzhou Tianhe Sports Centre Stadium	0.404	Slightly suitable
35	Hui Jing Golf Club	0.403	Slightly suitable
36	Lanpeng Golf Training Centre	0.443	Slightly suitable
37	Guangzhou Players Karting Club	0.454	Suitable
38	Seventy-two Par Golf Club	0.462	Suitable
39	Sports East Football Stadium	0.470	Slightly suitable
40	Bayi Football Team	0.449	Slightly suitable
41	Lifang Sports Wisdom Football Park	0.447	Slightly suitable
42	Nanqing Football Club	0.454	Slightly suitable
43	MVP Football Pitch (Zhujiang New Town Shop)	0.441	Slightly suitable
44	Tongdong Football Ground	0.460	Suitable
45	Tianhe Village Football Pitch	0.473	Suitable
46	Utopia Vitality Base Vitality Football Pitch	0.477	Suitable
47	Back Garden Tennis Club	0.458	Suitable
48	Juntai Tennis Centre	0.472	Suitable
49	Guangzhi Sports Basketball Centre	0.462	Suitable
50	Fei Meng Basketball Park (Botanical Garden)	0.456	Suitable

Suitable (R ≥ 0.45): There are 21 “suitable” emergency shelters in the study area, including two parks (South China Botanical Garden and Tianhe Park), eight squares (Pearl River Main Stream Square, Sports East Leisure Belt Square, etc.), and 11 sports venues (Guangdong Olympic Sports Center, Guangzhou Sports Basketball Hall, etc.). These shelters are suitable for good use and have reasonable spatial layouts, sizeable open space ratios, large practical shelter areas, large numbers of people, high road density, and slight slopes. When disasters occur, it can meet the needs of more victims. The roads are dense and can accommodate a large number of people.

Relatively suitable (0.40 ≤ R < 0.45): There are 25 “relatively suitable” emergency shelters in the study area, including 14 parks, such as Pearl River Park and Tianhe Wetland Park, five squares, such as Chebei Square and Guangzhou East Railway Station Square, and six sports venues, such as Guangzhou Tianhe Sports Center Stadium and Bayi Football Team. The decision-making value R of these shelters is larger, the distance from the nearest fire station is smaller, and the distance from the significant hazard source is more extensive, so the suitability is better. However, due to the low population capacity, the extended distance from medical stations and public security organs, and the high height of surrounding buildings, shelters' service is poor, reducing their suitability.

Unsuitable (R < 0.40): There are four emergency shelters at the “unsuitable” level in the study area, which are mainly parks, including Yinpailing Park, Carambola Park, Changyuan Park, and Changxin Village Garden. Among them, the main reasons affecting Yinpailing Park are that it is far away from the nearest medical station and the nearest public security organ, the slope is large, and the distance from significant hazards is considerable; that is, the accessibility and safety of the shelter are relatively low, which leads to the unsuitable level of the shelter. However, the distance between Changxin Garden and the nearest fire station is relatively far, and the number of people that can be accommodated is low, so its suitability is poor; the road density of Changxin Garden is low, and the distance between Changxin Garden and the nearest fire station is far, so the accessibility index is low, so its suitability is not high; the height of buildings around Carambola Park is high, and the distance between Changxin Garden and the nearest medical station is far, so its suitability is not high.

### 5.2 Optimization of the spatial layout of emergency shelters in Tianhe District

**Fig. 4:**
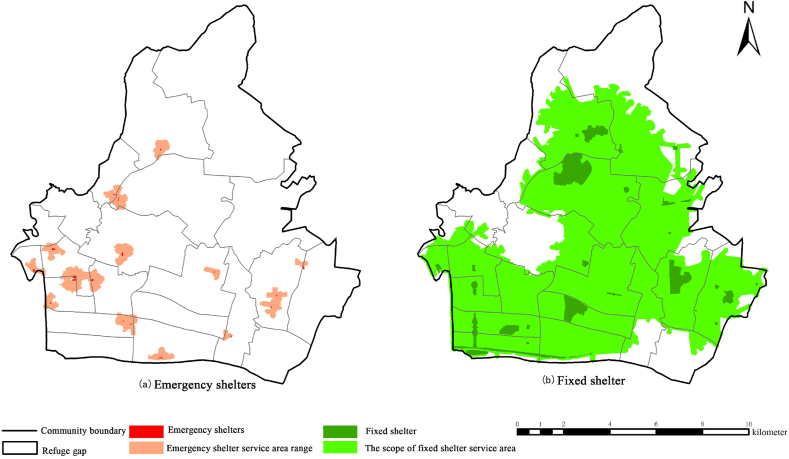
Coverage of service areas of emergency shelters in Tianhe District.

The overall suitability of emergency shelters in Tianhe District is suitable, but some emergency shelters are still unsuitable. Using ArcGIS network analysis, four unsuitable shelters were eliminated, and 500 m and 2500 m were used as the service radius of emergency shelters and fixed shelters to analyze the service area of emergency shelters in Tianhe District (Yan et al., 2010) [33]. According to the comprehensive analysis of Fig. 4a-4b, it was found that the existing suitable and more suitable shelters covered the central built-up area and the southwest commercial center gathering area of Tianhe District. The coverage rate of the service area is 68.4 %, but there is still a large shelter gap, which can not meet the shelter needs of residents in Tianhe District.

**Fig. 5:**
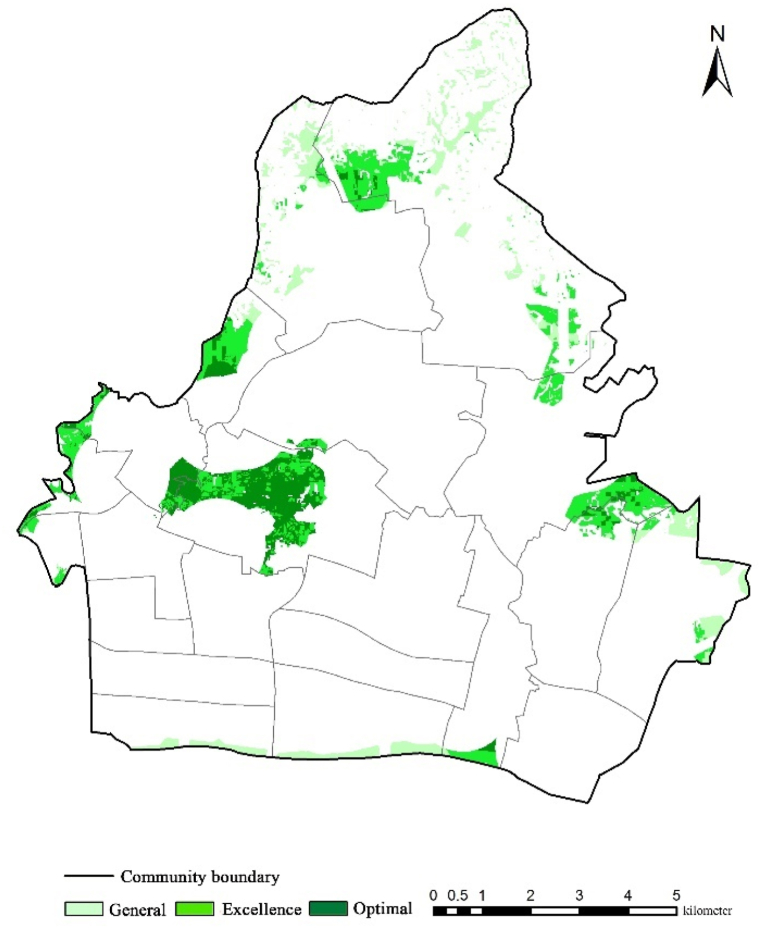
Alternative regional levels for emergency shelter.

The index connotation and scoring basis of each evaluation factor are summarized according to the relevant provisions on the site selection of emergency shelters in the Site Selection and Supporting Facilities of Earthquake Emergency Shelters (GB21734-2008) and other specifications and concerning the index scoring method [27] in the relevant domestic literature (Table 7). Combined with the research literature that many scholars at home and abroad have applied the analytic hierarchy process to evaluate the emergency shelters, the value assignment is carried out (Table 8) (Huang et al., 2006; Liu et al., 2010; Chu & Su, 2006) [[34], [35], [36]]. Finally, in the overlay analysis of ArcGIS, the comprehensive score is calculated by assignment, and the layout optimization scheme of the optimal location is obtained. According to the composite score, alternative regional levels for emergency shelter are divided into three: the general、the excellent、the optimal (Fig. 5).

**Table 7:** Scoring of emergency shelter layout optimization index.

Target layer	Criterion layer	Indicator name	Scoring basis	Score
Layout Optimization of Emergency Shelter	Fairness	Refuge demand point	The population density is more than 20000.	5
			Population density within 2000–20000 people	3
			The population density is below 2000.	1
	Accessibility	Road density	Road density above 30	5
			Road density within 10–30	3
			Road density below 10	1
		Distance from the nearest medical station	Within 500m from the nearest medical station	5
			Within 500–2000 m from the nearest medical station	3
			Distance to the nearest medical station: more than 2000 m	1
		Distance from the nearest fire station	Within 500m from the nearest fire station	5
			Within 500–2000 m from the nearest fire station	3
			More than 2000 m from the nearest fire station	1
		Distance from the nearest public security organ	Within 500m from the nearest public security organ	5
			Within 500–2000 m from the nearest public security organ	3
			More than 2000 m away from the nearest public security organ	1
	Security	Height of surrounding buildings	The height of surrounding buildings is below 15m	5
			Height of surrounding buildings within 15–25 m	3
			The surrounding buildings are more than 25m high.	1
	Feasibility	Land use status	The current situation of land use is bare land and pasture.	5
			The current land use situation is a combination of trees and built-up areas.	3
			The current land use situation is water bodies, flooded plants, and crops.	0

**Table 8:** Weighting values of elements.

Objective level	Criterion level	Primary weights	Indicator level	Secondary weights
Optimization of the layout of emergency shelters	Fairness	0.2978	Points of Refuge Needs	0.2978
	Accessibility	0.4195	Road density	0.1533
			Distance to nearest medical site	0.1258
			Distance to the nearest fire station	0.0851
			Distance to the nearest public security organ	0.0553
	Security	0.1861	Height of surrounding buildings	0.1861
	Feasibility	0.0965	Land use status	0.0965

**Fig. 6:**
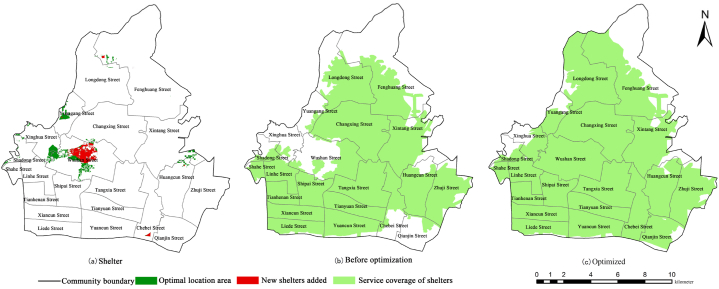
New emergency shelters, coverage of emergency shelter service area before and after optimization.

The urbanization development of Tianhe District is relatively fast, and there may be a lack of reserved land in the city. Therefore, according to the optimal site selection distribution, combined with the image map, the optimal site selection area of Tianhe District can be implemented in schools, parking lots, pastures, and open land to achieve the principle of “dual use of peace and disaster” and reduce the pressure of land development. Finally, the optimal site selection area of more than 300 ha is implemented for five new emergency shelters, and the layout optimization of supplementary site selection is carried out, as shown in Table 9 and Fig. 6a - c.

**Table 9:** Information on new emergency shelters.

Location	Site Specific Location	Type of Evacuation	optimal area/hm^2^	Optimum effective evacuation area/hm^2^	Population capacity/people
West	South China Agricultural University	Fixed Emergency Shelter	117.77	58.89	294425
	Yuen Kong Street Car Park	Temporary Emergency Shelter	0.47	0.47	4700
North	Pastureland between Fisherman's West Road and South China Express Trunk Road	Fixed Emergency Shelter	2.67	2.67	13350
East	Pastureland next to Hopson Imperial Villa	Temporary Emergency Shelter	0.59	0.59	5900
Southeast	Open space to the west of Chepi Chung Hau	Temporary Emergency Shelter	4.26	4.26	21300

According to the calculation and visualization results, it can be seen that the location of new shelters in the alternative areas is mainly located in the west of Tianhe District, followed by the eastern and southeastern marginal areas, which conforms to the principles of fairness, accessibility, safety, and feasibility, and the road density is high, most of them are built-up areas, and the most critical factor is the excellent combination with the distribution of population density. Reflect the core principle of people-oriented site selection of the shelter; the northern part of Tianhe District is mainly low mountains and hills with steep slopes, and the land type is mainly trees and forests, and there is no distribution of residential areas, so the cost performance of establishing the shelter is low and the risk is high, so it is not suitable to be used as the site selection area of the new shelter; The western and southeastern edge areas of Tianhe District also have most of the dangerous areas within 1000 m from the gas station, so they are not suitable for the location of new shelters. According to the optimal site selection area statistics, there is a total of 322.81 hm ^2^, while the number of people in the shelter gap is 387,900. According to the calculation of the per capita effective shelter area of emergency and fixed shelters, about 38.79 hm ^2^ and 77.58 hm ^2^ are needed, far less than the optional area of the optimal site selection. In general, the optimal site selection area meets the shelter needs of the study area.

**Fig. 7:**
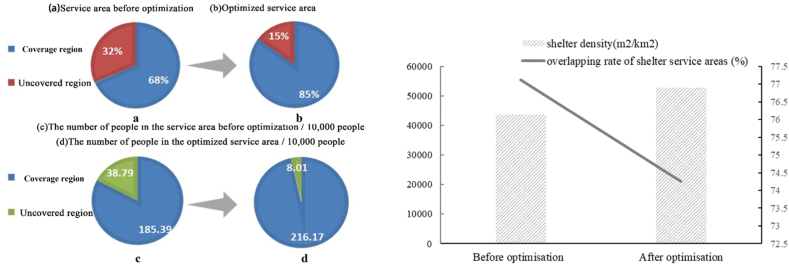
Changes in relevant indicators of emergency shelters before and after optimization.

According to Table 8, the number of people served by the new shelters in the service area is 107,385 people at South China Agricultural University (excluding the students of the university), 6,420 people in the parking lot of Yuangang Street, 3,563 people in the northern grassland 11465, 3,563 people in the eastern grassland, and 180713 people in the open space in the west of Chebei Chung. Combined with Table 8, it is found that the space on the new shelters of South China Agricultural University, the northern grassland, and the eastern grassland can meet the number of residents in their service areas. However, the space in the parking lot of Yuangang Street and the newly built shelter in the open space on the west side of Chebei Chung cannot meet the number of residents in its service area. Among them, the supply-demand relationship in the open space on the west side of Chebei Chung is still tense, and the number of people needing shelter is more than eight times the number of people that can be accommodated. Still, on the whole, it also alleviates the demand for shelter in the southeast of Tianhe District. Through the addition of five new emergency shelters, the capacity of Tianhe District to respond to and prevent disasters has been greatly enhanced. As can be seen from Fig. 7, the emergency shelters in Tianhe District could only cover 68 % of the area, with 1,853,900 people in the service area before optimization. After optimization, the emergency shelters in Tianhe District covered 85 % of the area, an increase of 17 %, which reduces the blind spot of the service; and at the same time the number of people in the service area has increased to 2,167,000, an increase of 307,900 people. At the same time, the density of the optimised emergency shelters (52722.4 m^2^/km^2^) is higher than that of the pre-optimization one (43604.6 m^2^/km^2^), which reduces the service blind zones; the service overlap rate can evaluate the fairness of the residents' actual enjoyment of the emergency shelters (Liu et al., 2012) [37], and it is found that overlapping rate of shelter service areas has been reduced to a certain extent by the addition of the five new emergency shelters, but the overlapping rate of shelter service areas in Tianhe District's is generally high, mainly because Tianhe District is the centre of Guangzhou City, with high population density, coupled with the overly dense layout of emergency shelters in some areas. In terms of quantity, density, number of people accommodated, service area, etc., the overall layout optimization scheme was more reasonable and a more complete system of emergency shelters has been formed, which is also an important part of the future promotion of the development of emergency management in Tianhe District.

## 6 Discussion

This paper explores the suitability evaluation and spatial layout optimization of urban emergency shelters. Still, due to limitations in data collection and time constraints, research scope, data collection and processing, and spatial layout optimization were not as thoroughly considered as they could have been, leaving some limitations. This study only considers the service facilities and hazards within the Tianhe District and excludes those near the border of the district. It was found that the open space ratio significantly contributes to suitability, suggesting that the calculation of the effective refuge area for emergency shelters should be more refined to minimize errors. When considering the demand for refuge, the supply and demand relationship of shelter has only been evaluated based on the capacity of the existing emergency shelters. The analysis did not refine the supply and demand relationship between the refuge population within the service scope of each shelter and its capacity. It is also necessary to analyze the supply and demand of each shelter within its service scope. These aspects need to be addressed to improve the spatial layout and functional optimization of emergency shelters.

Emergency shelters are essential for the construction of resilient cities and should withstand the challenges of various types of disasters. The site selection and construction of emergency shelters should be more people-oriented by considering residents' needs and enhancing the comprehensiveness of emergency shelters for catastrophes. Future research should further clarify the differences in the safety requirements for shelters targeting different disasters and consider the needs of victims in other disaster scenarios (Li & Fu, 2017) [38]. Urban resources and funds are limited, making it impractical to build shelters repeatedly (Ding et al., 2015) [39]. Therefore, it is of great practical significance to study comprehensive shelter planning for multi-disaster scenarios. Future studies should focus on emergency shelters based on earthquake prevention and include a wider range of disaster indicators to comprehensively evaluate the suitability of emergency shelters. Finally, spatial layout optimization should consider not only the current land use, but the city's development planning to determine the optimal scheme. At present, China's emergency shelters and public security risk assessment are mostly focused on single disasters and accidents, and the type refinement, spatial scale and functional optimization of emergency shelters under multi-hazard response and coupled disaster system scenarios can be further improved, and the development of regional multi-hazard emergency shelter spatial pattern and functional optimization frameworks and systems is of great significance to the regional multi-hazard comprehensive risk assessment. This is also an important direction for our subsequent research.

## 7 Conclusion and recommendations

### 7.1 Conclusion

Urban emergency shelter is an important factor in the construction of urban disaster prevention capability and resilient city. This paper takes the earthquake emergency evacuation space in Tianhe District of Guangzhou City as the research object, selects the three indicators of effectiveness, accessibility and safety to construct the suitability evaluation index system, and applies the grey correlation method and entropy weighting method to calculate the suitability of each existing evacuation space. Based on the evaluation results, unsuitable places of refuge were excluded and evacuation gaps were extracted. To determine the alternative areas for the evacuation gaps, through the principles of fairness, accessibility, safety and feasibility, and through the combination of AHP hierarchical analysis and ArcGIS technology, the optimal location of the alternative areas is determined, and the spatial layout optimization plan for emergency evacuation sites in Tianhe District is proposed. The research of this paper is an attempt to the comprehensive method of spatial optimization of emergency refuge places, and also provides relevant theoretical support and case studies for the planning and layout of emergency refuge places in Tianhe District of Guangzhou and related city types across the country. The following conclusions are obtained.

(1) There is a significant difference in the weight distribution of the suitability rating indicators of emergency shelters in Tianhe District: the number of people that can be accommodated contributes the most to the suitability evaluation results, accounting for more than half of the total weight. The weight of road density under the accessibility principle is also substantial, while the contribution of other indicators is small. Among the safety indicators, the weight of the distance from significant hazard sources is the largest. These provide a relevant foundation for the construction and management of emergency shelters in Tianhe District.
(2) The overall suitability of emergency shelters in Tianhe District is acceptable: the suitability evaluation results are divided into the following three grades: suitable (R ≥ 0.45); relatively suitable (0.40 ≤ R < 0.45); and unsuitable (R < 0.40). Twenty-one emergency shelters, including South China Botanical Garden, Pearl River Mainstream Square, and Guangdong Olympic Sports Center are at the “suitable” level; Twenty-five emergency shelters, such as Pearl River Park, Guangzhou East Railway Station Square, and Guangzhou Tianhe Sports Center Stadium, are at the “relatively suitable” level; Four emergency shelters, including, Yinpailing Park, Carambola Park, Long Park and other four emergency shelters, are at the “unsuitable” level.
(3) Most of Tianhe District's main shelters are far from the district's center. The shelter space in Tianhe District has been reasonably allocated by optimizing the original layout. An analysis of the service area indicates that the service coverage rate of the existing shelters in Tianhe District is only 68.4 %, leaving 387,900 people without shelter. The location grades of the emergency shelters in Tianhe District are divided into three levels. The results show that the optimal locations for new shelters are primarily in the west and southeast of Tianhe District, which is aligned with the distribution of population density. According to the optimal location distribution and the image map, the layout optimization scheme was implemented from the optimal location area to five new emergency shelters. The space at South China Agricultural University, the northern grassland and the eastern grassland can accommodate the residents in their service areas. However, the space at the parking lot of Yuangang Street and the open space on the west side of Chebei Chung cannot meet the needs of the residents in their service area. However, the overall optimization has alleviated the demand for refuge in Tianhe District. After the layout optimization, the service scope of shelters in Tianhe District covers residential areas with a coverage rate of 85. 2 %, providing refuge security for 307,800 people, and the overlapping rate of shelter service areas has been reduced to a certain extent by the addition of the five new emergency shelters. Overall, the layout optimization scheme is more reasonable. These provide recommendations for the post-planning of emergency shelters and the construction of resilient cities in Tianhe District.

### 7.2 Recommendation

(1) Upgrading the level of suitability of emergency shelters, optimize and adjust the existing shelters. The spatial distribution of emergency shelters in Tianhe District varies widely, affecting the overall disaster response capacity. In the southeast refuge gap, the number of people in need of refuge is large, so it is suggested to optimize the accessibility and safety indicators that lead to the poor suitability of the carambola park, increase the service facilities near the carambola park, reinforce the high-rise buildings around the carambola park, and relocate the gas station, so as to enhance the suitability of the carambola park as a shelter, so as to reduce the waste of resources. It also helps to alleviate the pressure of population demand in the surrounding shelters.
(2) As the suitability of emergency shelters in Tianhe District is closely related to the number of people they can accommodate and their distance from major sources of hazards. On the one hand, on the basis of land use planning, appropriate alterations, expansions and new emergency shelters are carried out to meet the sheltering needs of more residents; on the other hand, properly relocate the gas station. A large number of open spaces beside Chebei Yongkou are also not suitable for site selection because they are located in a dangerous construction area 1000 m away from the gas station. Gas station, as a hazard factor, greatly affects the suitability and location of shelters in the southeast of Tianhe District. In order to alleviate the pressure of shelter demand, it is a cost-effective scheme to relocate the gas station. In the west of Tianhe District, there is still a large number of people in need of refuge who are not covered by the refuge service area, and most of the sites are located in dangerous construction areas, so it is suggested to relocate the gas station.
(3) Although the optimised programme has contributed to the emergency sheltering and disaster prevention and mitigation capacity of Tianhe District, there are still some sheltering gaps and service blind spots, and the service overlap rate is relatively high. Therefore, emergency shelters in Tianhe District need to be constructed in accordance with the planning and relevant standards, forming a mode of ‘government-led, hierarchical responsibility and multi-body participation’ to enhance the efficiency of emergency management. For example, the relevant departments (units) of the district government are responsible for the daily maintenance and management of emergency shelters; the streets (towns) to the community (village) as a unit, drawing the distribution of emergency shelters within the jurisdiction of the distribution of emergency shelters, the scope of services and the surrounding public emergency evacuation roadmap. At the same time, relying on the emergency shelter to carry out emergency publicity and education activities to enhance the awareness of disaster prevention and mitigation throughout the community and to improve the public's ability to emergency evacuation and self-rescue and mutual aid.

## CRediT authorship contribution statement

**Bo Tang:** Writing – review & editing, Resources, Funding acquisition, Formal analysis, Data curation. **Zongyuan Li:** Methodology, Investigation, Formal analysis, Data curation. **Yinzhong Chen:** Software, Formal analysis.

## Funding

This work was supported by Philosophy and Social Science Planning Project of Guangdong Province (No. GD24XGL035); Guangdong General Colleges and Universities Characteristic and Innovative Projects (No. 2024KTSCX126)；Teaching Reform Project of Guangzhou Xinhua College (No. 2023KCJ001); College students’ innovative entrepreneurial training program (NO. 202413902003).

## Data availability statement

Data included in article/supplementary material is referenced in the article.

## Declaration of competing interest

The authors declare the following financial interests/personal relationships which may be considered as potential competing interests:BoTang reports financial support was provided by School of Resources and Planning, Guangzhou Xinhua University. If there are other authors, they declare that they have no known competing financial interests or personal relationships that could have appeared to influence the work reported in this paper.

## References

[1] Wang S.Y. (2003). Constructions of emergency management capability to urban disasters. City Disaster Reduct..

[2] Fang C.L., Wang Y. (2015). A comprehensive assessment of urban vulnerability and its spatial differentiation in China. Acta Geograph. Sin..

[3] Zhai G.F., Xia C.H. (2021). Strategic emphasis on the construction of resilient cities in China. City Plann. Rev..

[4] Ming X.D., Xu W., Liu B.Y., Du J., Ku Z.W., Ge Y. (2013). Research progress in multi-hazard risk assessment. J. Catastrophol..

[5] Zhao L.J., Wang K., Wang J. (2014).

[6] Marjolein S., Bas W. (2017). Building up resilience in cities worldwide-Rotterdam as participant in the 100 resilient cities programme. Cities.

[7] Bandana K., Michael E.H. (2008). A GIS‐based model to determine site suitability of emergency evacuation shelters. Trans. GIS.

[8] Chou J., Ou Y., Cheng M.L. (2013). Emergency shelter capacity estimation by earthquake damage analysis. Nat. Hazards.

[9] Chen Z.F., Zhou J., Wang J.Z., Zou L., Xie Y.X. (2016). A simple way to predict evacuation populations in emergency shelter planning: exemplified by the earthquake disaster. City Plann. Rev..

[10] Anhorn J., Khazai B. (2015). Open space suitability analysis for emergency shelter after an earthquake. Nat. Hazards Earth Syst. Sci..

[11] Akamatsu T., Yamamoto K. (2019). Suitability analysis for the emergency shelters allocation after an earthquake in Japan. Geosciences.

[12] Yao Y., Zhang Y., Yao T., Wong K., Tsou J.Y., Zhang Y. (2021). A GIS-based system for spatial-temporal availability evaluation of the open spaces used as emergency shelters: the case of Victoria, British Columbia, Canada. ISPRS Int. J. Geo-Inf..

[13] Liu Y.Q., Hu Q.W., Cheng G., Yue J.S. (2017). Research on suitability evaluation method of urban shelters based on gray relational analysis and entropy weight. J. Wuhan Univ. Technol. (Inf. Manag. Eng.).

[14] Wang X., Guan M., Dong C., Wang J., Fan Y., Xin F., Lian G. (2022). A multi-indicator evaluation method for spatial distribution of urban emergency shelters. Rem. Sens..

[15] Tang B., Qiu F.P., Huang J.Y. (2017). Research progress of emergency shelter in China from the perspective of resilient city. Modern Urban Res..

[16] Constantine T., Ralph S., Charles R., Lawrence B. (1971). The location of emergency service facilities. Oper. Res..

[17] Wu W.J., Zhu S.Y., Zhang W.Z. (2010). Optimal allocation of emergency shelter facilities in Beijing. Hum. Geogr..

[18] Zhou A.H., Zhang J.Q., Fu X. (2014). On the spatial distribution of the urban-emergency shelter in Beijing downtown areas. J. Saf. Environ..

[19] Trivedi A., Singh A. (2017). A hybrid multi-objective decision model for emergency shelter location-relocation projects using fuzzy analytic hierarchy process and goal programming approach. Int. J. Proj. Manag..

[20] Li Y.L., Chen T., Zang X.Y. (2018). GIS-supported research on urban earthquake emergency response capacity. World Earthq. Eng..

[21] Tang B., Qiu J. (2019). Knowledge structure of emergency shelters research: an analysis based on WoS and Citespace map. World Reg. Stud..

[22] Wu W.B., M J., Banzhaf E., Meadows M.E., Yu Z.W., Guo F.X., Sengupta D., Cai X.X., Zhao B. (2023). A first Chinese building height estimate at 10 m resolution (CNBH-10m) using multi-source earth observations and machine learning. Remote Sens. Environ..

[23] Shi T.G., Zheng G.Q., Wang Z.Y., wang L.L. (2007). Progress in research on land suitability evaluation in China. Prog. Geogr..

[24] Huang Y., Luo Z.Y., Yang W.N. (2008). Urban dwelling feasibility evaluation research based on GIS. Sci. Surv. Mapp..

[25] Tie Y.B., Tang C. (2005). Establish the evaluation system of urban disaster emergency response capability. Urban Problems.

[26] Lv W., Han Y.F., Zhou W.N., Shi S.L. (2023). Study on the spatial correlation between the suitability evaluation of emergency shelters and the service scope. J. Saf. Environ..

[27] Liu Y.B., Li R.D., Song X.F. (2005). Grey associative analysis of regional urbanization and eco-environment coupling in China. Acta Geograph. Sin..

[28] Yu X.F., Fu D. (2004). An overview of multi-indicator integrated evaluation methods. Stat. Decis..

[29] Zhang S., Zhang M., Chi G.T. (2010). The science and technology evaluation model based on entropy weight and empirical research during the 10th five-year of China. Chin. J. Manag..

[30] Ji W.J., Qu J.S., Xu L. (2021). Disaster mitigation capacity evaluation of emergency shelters of Lanzhou city based on AHP and GIS techniques. Remote Sens. Technol. Appl..

[31] James G. (2008). Dolan. Shared decision-making–transferring research into practice: the Analytic Hierarchy Process (AHP). Patient Educ. Counsel..

[32] Li Y.L., Wang P.M., Cheng S. (2015). Network analysis method of fixed emergency shelters layout evaluation. World Earthq. Eng..

[33] Yan Y., Tang Y., Wei Z. (2010). Chongqing Municipal People's Government. Planning Innovation: Proceedings of the 2010 Annual Conference on Urban Planning in China.

[34] Huang D.J., Wu Z.Z., Cai S.J., Jiang Z. (2006). Emergency adaption of urban emergency shelter:analytic hierarchy process-based assessment method. J. Nat. Disasters.

[35] Liu Q., Ruan X.J., Fu B.H. (2010). A study of principles on emergency shelter site selection and modeling under disastrous earthquake. Period. Ocean Univ. China.

[36] Chu J.Y., Su Y.P. (2006). The planning principles and requirements of urban seismic refuges for evacuation. World Earthq. Eng..

[37] Liu S.L., Y Q.Lu, Gu X.P., Pei Y.F., Liu T. (2012). Reasonability of spatial distribution for urban emergency shelter. Urban Dev. Stud..

[38] Li C.Y., Fu J.L. (2017). A review of service capacity evaluation of urban emergency shelters. J. Inst. Disaster Prev..

[39] Ding L., Zhai G.F., Li S.S. (2015). Planning for urban comprehensive evacuation shelters for better response to disasters. City Plann. Rev..

