# Supplementary material for: Spatial suitability evaluation and layout optimization of emergency shelter: A case study in Tianhe District of Guangzhou City
Source: Heliyon. 2024 Dec 12;11(1):e41122. doi: 10.1016/j.heliyon.2024.e41122 (PMC11728944; doi:10.1016/j.heliyon.2024.e41122)
Supplement: Multimedia component 1 [file mmc1.docx]

Supplementary data mainly includes road density(Fig.8)、population density (Fig.9)、land use types (Fig.10)、elevation(Fig.11)、buildings outline（Fig.12）、 buildings height (Fig. 13)、distribution of facility sites (Fig. 14(a - d))、nearest route from emergency shelters to facility sites (Fig. 15(a - c)).


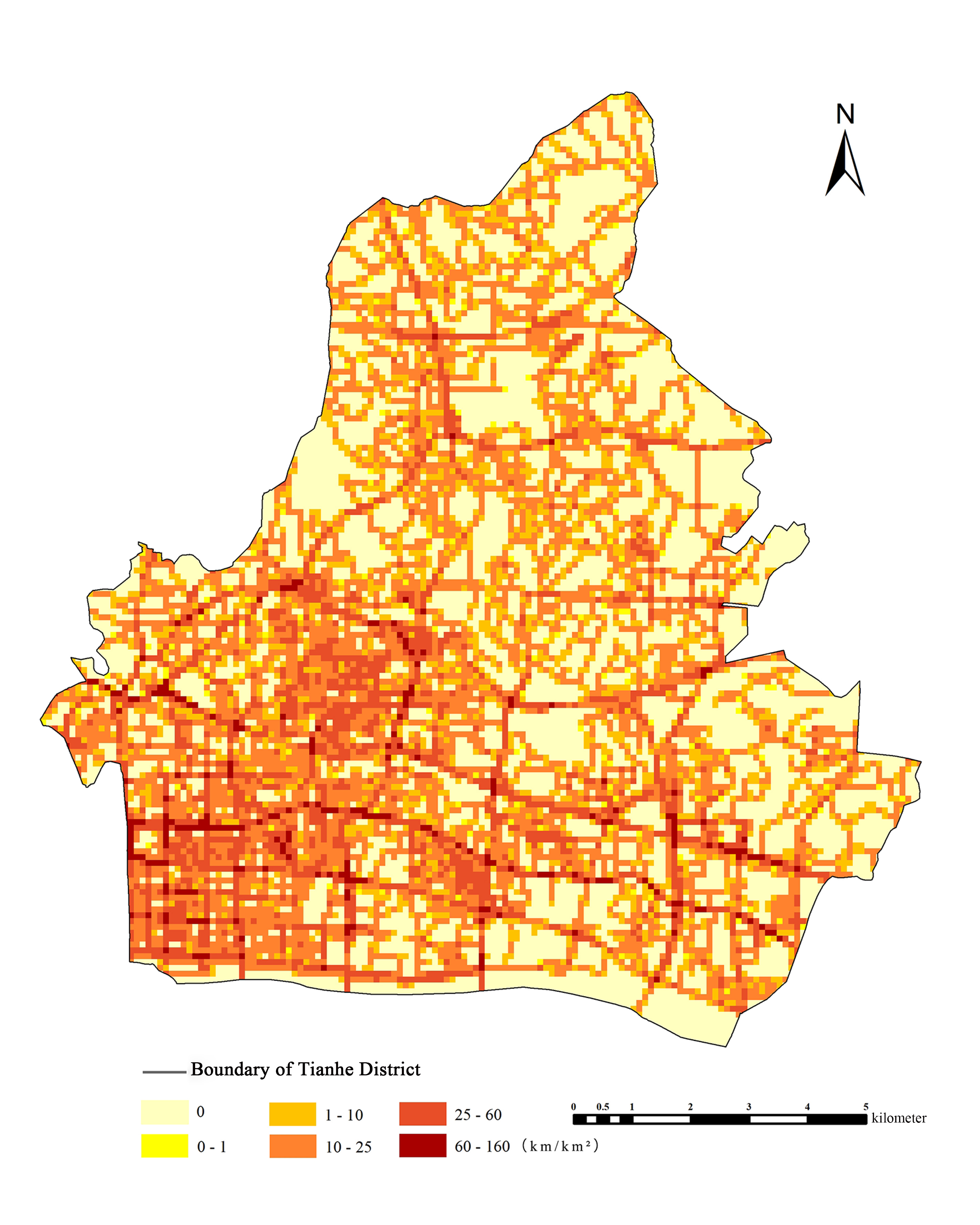

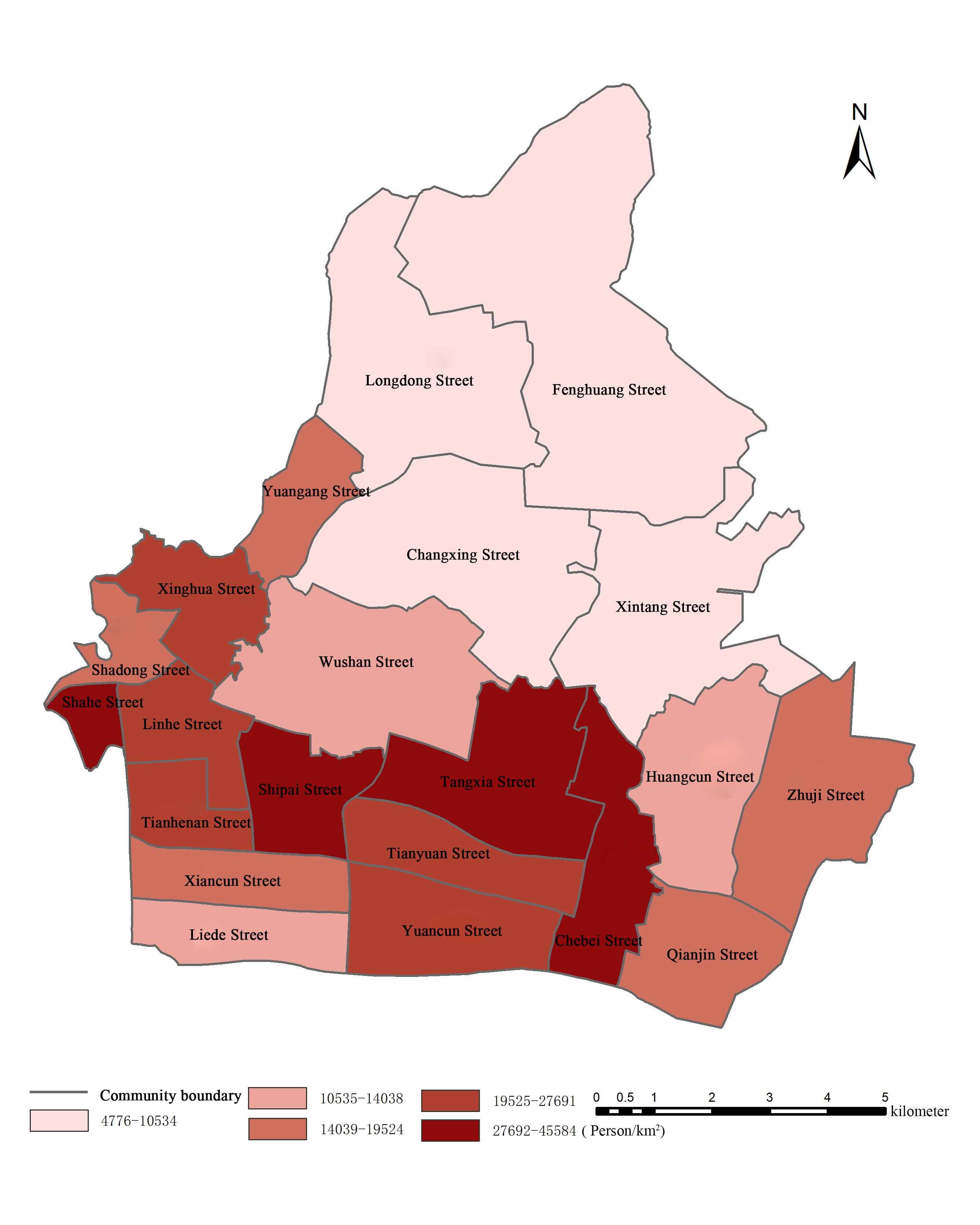


Figure 8 Road Density Figure 9 Population Density


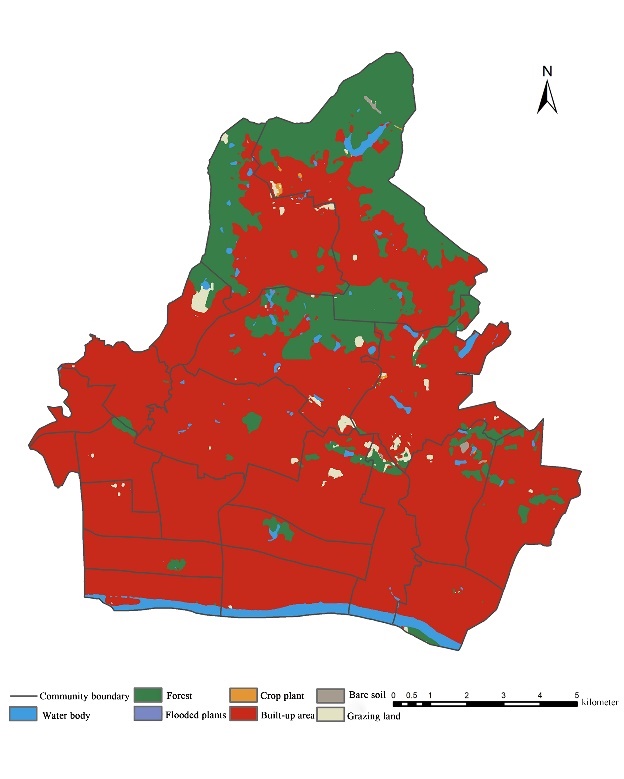

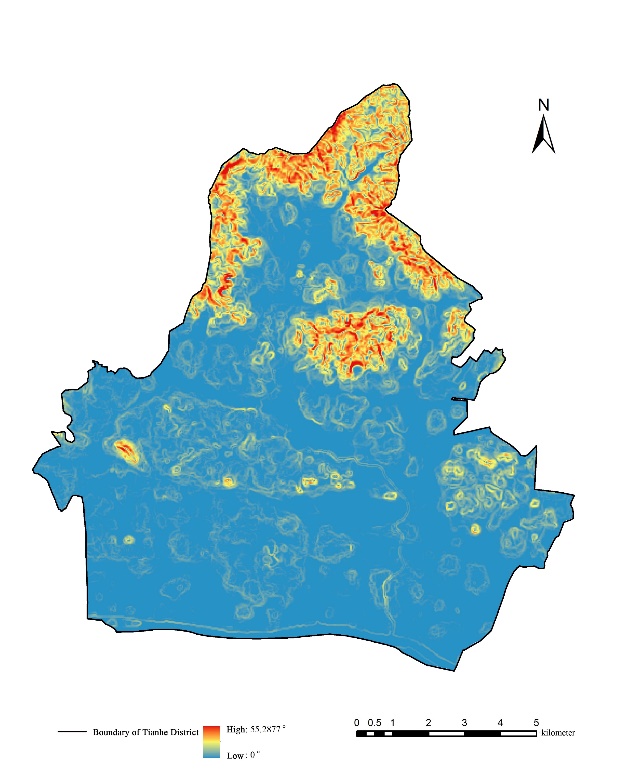


Figure 10 Land Use Types Figure 11 Elevation


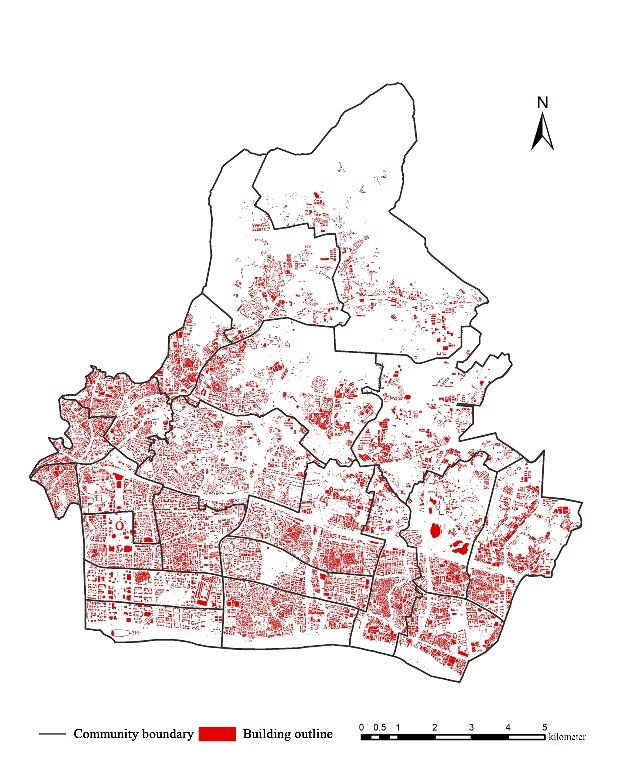

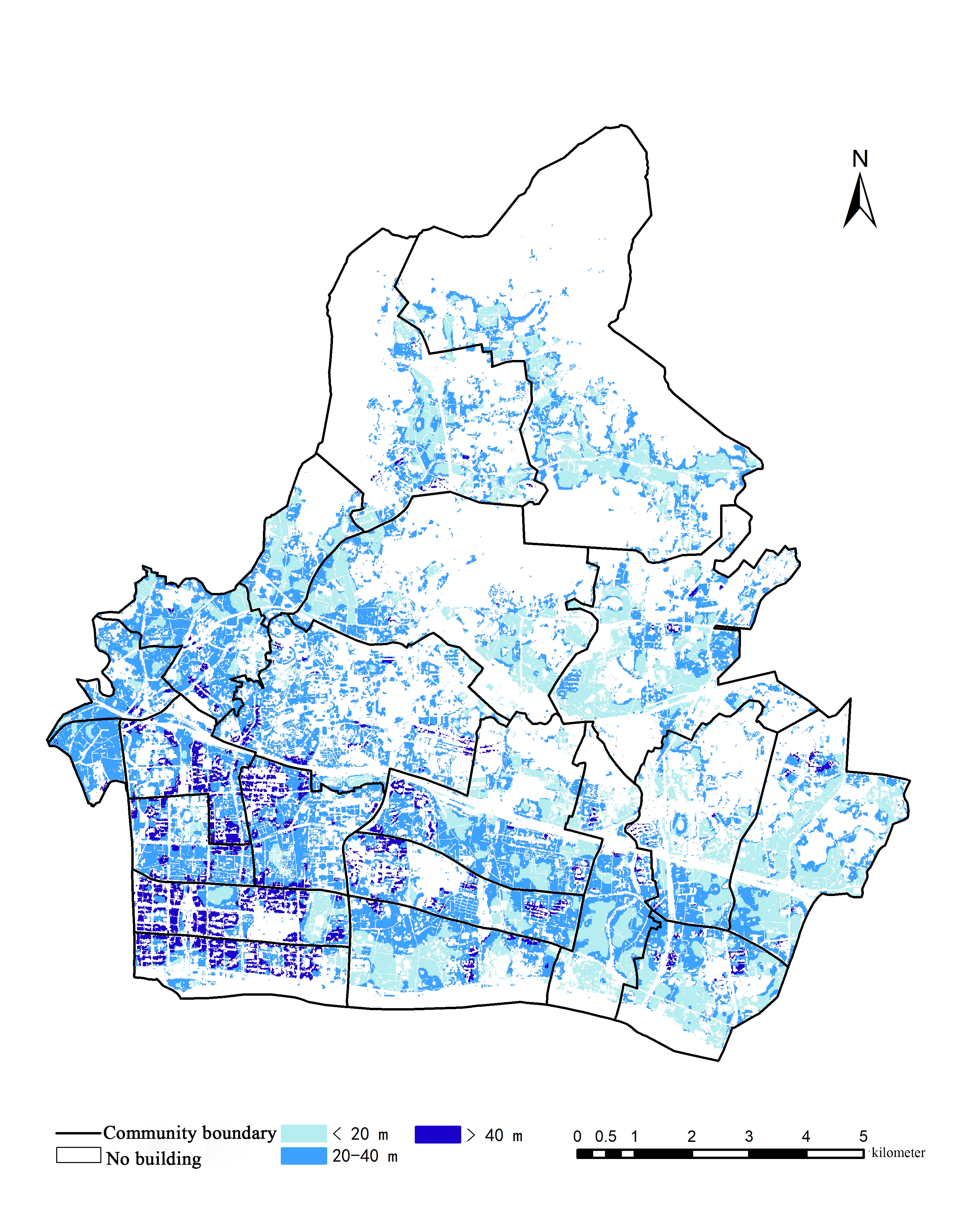


Figure 12 Buildings Outline Figure 13 Buildings height


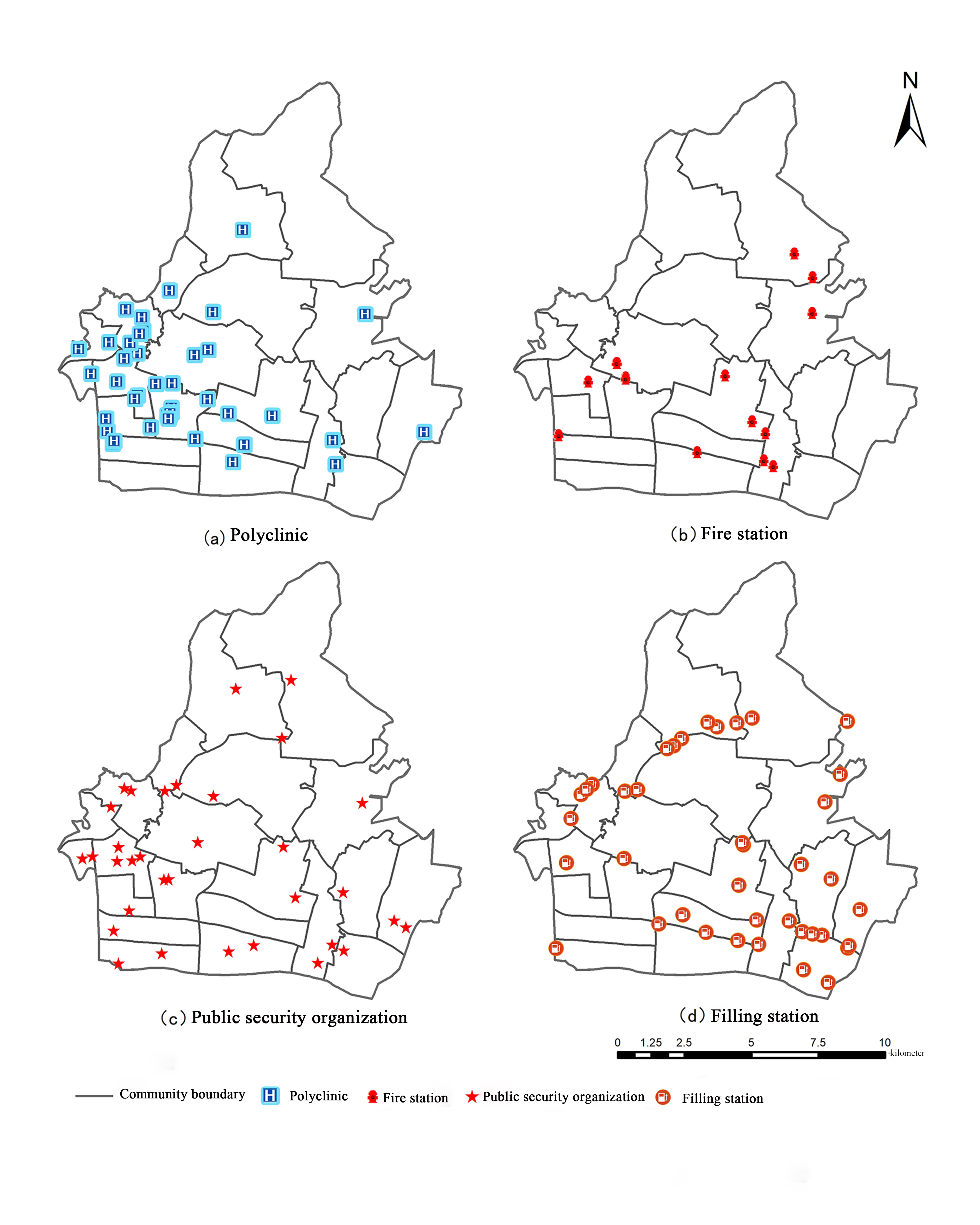


Figure 14 Distribution of Facility Sites


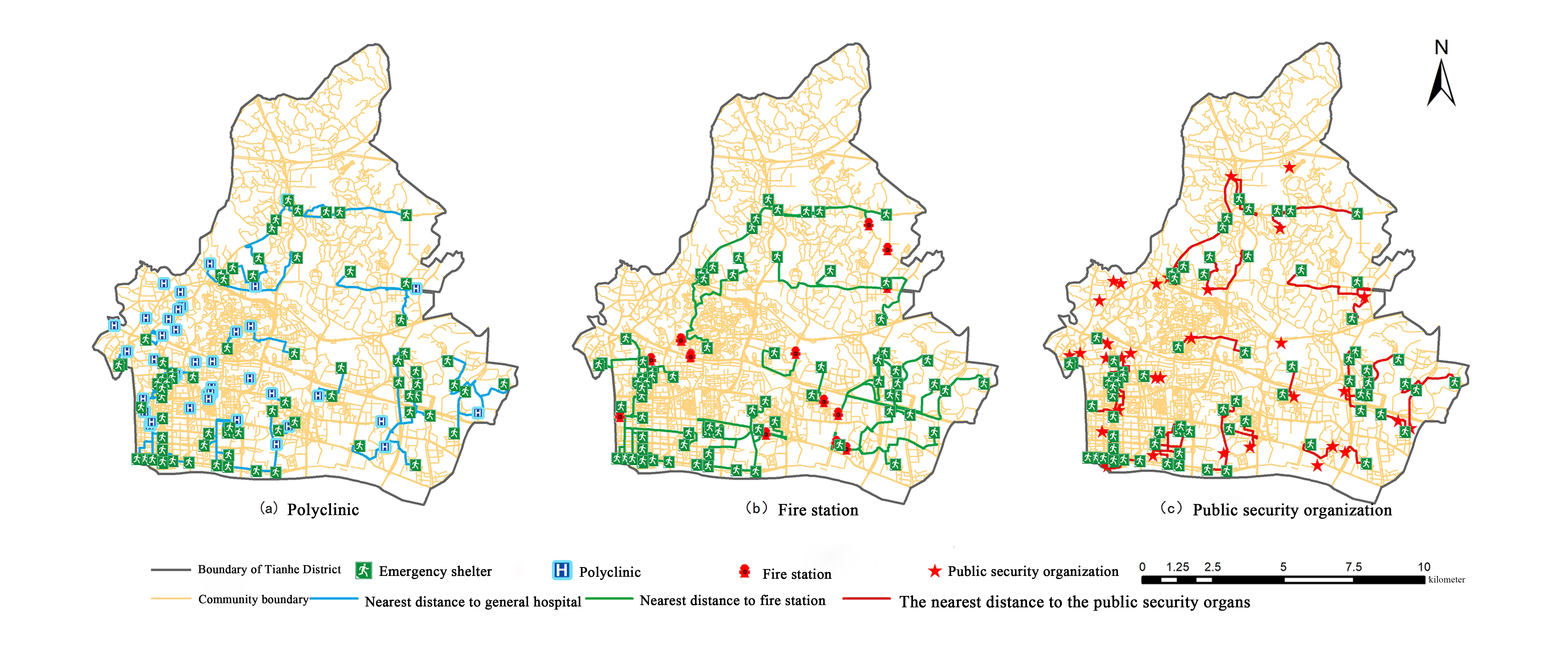


Figure 15 Nearest route from emergency shelters to facility sites
